# Retraction: Multi-scale Inference of Interaction Rules in Animal Groups Using Bayesian Model Selection

**DOI:** 10.1371/annotation/7bc3a37e-db82-4813-8242-7d34877125c5

**Published:** 2012-08-07

**Authors:** Richard P. Mann, Andrea Perna, Daniel Ströbom, Roman Garnett, James E. Herbert-Read, David J. T. Sumpter, Ashley J. W. Ward

*The authors are retracting this paper. The first author explains the reasons below:*

A bug was found in the Matlab code used in this study, which resulted in only a small proportion of the full data set being analysed. Where each of 102 experiments should have been down-sampled to half the original size for computational efficiency, instead the number of experiments in the data set was repeatedly halved 102 times, until only one remained. As a consequence of this, our results and conclusions were based on only one experimental study, rather than the 102 reported in the paper. 

After correcting the bug and reanalyzing the full data set we found that our results had changed significantly, and some of our conclusions were no longer valid. The empirically observed phase transition and collective behaviour remain, as does the observation that individuals are more likely to change direction when in close proximity to each other. However, the likelihood ordering of the different models for interactions between individuals is changed, and there is no longer a failure to reproduce large-scale results by simulation of the Markovian spatial models. In conjunction with the editors we therefore decided that the paper must be retracted. 

The responsibility for this coding error is entirely mine (Richard Mann). My coauthors were not involved in coding this stage of the analysis. I am grateful to Michael Osborne (University of Oxford) and David Duvenaud (University of Cambridge) who spotted this error when I passed the code and data on to them, while aiming to replicate our results for their own project.

We will be assessing the conclusions to be drawn from our reanalysis of the data and submitting a revised paper for publication in the future.

